# Supplementary material for: 3D reconstructions of parasite development and the intracellular niche of the microsporidian pathogen Encephalitozoon intestinalis
Source: Nat Commun. 2023 Nov 23;14:7662. doi: 10.1038/s41467-023-43215-0 (PMC10667486; doi:10.1038/s41467-023-43215-0)
Supplement: Supplementary file 3 — Description of Additional Supplementary Files [file 41467_2023_43215_MOESM3_ESM.pdf]

Description of Additional Supplementary Files

File Name: Supplementary Data 1
Description: Analysis and composition of parasitophorous vacuoles across all SBF-SEM datasets of infected Vero cells at 24 hpi and 48 hpi.

File Name: Supplementary Movie 1
Description: 3D reconstruction of a parasitophorous vacuole from an *E. intestinalis*-infected Vero cell.
Representative reconstruction of a parasitophorous vacuole (green) containing sporonts (blue).

File Name: Supplementary Movie 2
Description: 3D reconstruction of an *E. intestinalis* sporont.
Representative reconstruction of an *E. intestinalis* sporont. Each color represents an individual organelle: exospore (yellow), nucleus (blue), ER (green).

File Name: Supplementary Movie 3
Description: 3D reconstruction of an *E. intestinalis* stage 1 sporoblast.
Representative reconstruction of an *E. intestinalis* stage 1 sporoblast. Each color represents an individual organelle: exospore (yellow), nucleus (blue), ER (green), nucleation center (magenta), polar tube (purple), posterior vacuole (red).

File Name: Supplementary Movie 4
Description: 3D reconstruction of an *E. intestinalis* stage 2 sporoblast.
Representative reconstruction of an *E. intestinalis* stage 2 sporoblast. Each color represents an individual organelle: exospore (yellow), nucleus (blue), ER (green), nucleation center (magenta), polar tube (purple), posterior vacuole (red).

File Name: Supplementary Movie 5
Description: 3D reconstruction of an *E. intestinalis* stage 3 sporoblast.
Representative reconstruction of an *E. intestinalis* stage 3 sporoblast. Each color represents an individual organelle: exospore (yellow), nucleus (blue), ER (green), nucleation center (magenta), polar tube (purple), posterior vacuole (red).

File Name: Supplementary Movie 6
Description: 3D reconstruction of an *E. intestinalis* spore observed in the parasitophorous vacuole.
Representative reconstruction of an *E. intestinalis* spore. Each color represents an individual organelle: exospore (yellow), nucleus (blue), ER (green), nucleation center (magenta), polar tube (purple), posterior vacuole (red), anterior polaroplast (teal).

File Name: Supplementary Movie 7
Description: 3D reconstruction of an *E. intestinalis* spore purified from Vero cells.
Representative reconstruction of an *E. intestinalis* spore. Each color represents an individual organelle: exospore (yellow), nucleus (blue), ER (green), polar tube (purple), anchoring disc

(orange), anterior polaroplast (teal). Posterior vacuole could not be segmented accurately in this spore and is not shown in the 3D reconstruction.

File Name: Supplementary Movie 8
Description: Live-cell imaging of host mitochondria remodeling in an *E. intestinalis* infected Vero cell.
Time lapse video of host mitochondria fragmentation (red) in an *E. intestinalis* infected Vero cell corresponding to Figure 6E in the main text.

File Name: Supplementary Movie 9
Description: Live-cell imaging of host mitochondria remodeling in an *E. intestinalis* infected Vero cell.
Time lapse video of host mitochondria fragmentation (red) in an *E. intestinalis* infected Vero cell that undergoes cell division.

File Name: Supplementary Movie 10
Description: *E. intestinalis* life-cycle. The movie incorporates our findings in the context of what is known from the literature.

File Name: Supplementary Movie 11
Description: *Development of an E. intestinalis parasite.* The movie incorporates cell shape and size changes, as well as the development of individual organelles that could be annotated in SBF-SEM datasets.

File Name: Supplementary Movie 12
Description: Model of *E. intestinalis* polar tube development based on data from SBF-SEM reconstructions.

File Name: Supplementary Movie 13
Description: Modeling PT coiling.
A stiff tubing (analogous to the polar tube) coils within the confines of a bottle (analogous to the spore) when continuously threaded through the top.
