# Supplementary material for: 3D reconstructions of parasite development and the intracellular niche of the microsporidian pathogen Encephalitozoon intestinalis
Source: Nat Commun. 2023 Nov 23;14:7662. doi: 10.1038/s41467-023-43215-0 (PMC10667486; doi:10.1038/s41467-023-43215-0)
Supplement: Supplementary file 18 — Reporting Summary [file 41467_2023_43215_MOESM18_ESM.pdf]

nature portfolio

Corresponding author(s): Gira Bhabha

Last updated by author(s): Sep 15, 2023

Reporting Summary

Nature Portfolio wishes to improve the reproducibility of the work that we publish. This form provides structure for consistency and transparency in reporting. For further information on Nature Portfolio policies, see our Editorial Policies and the Editorial Policy Checklist.

Statistics

For all statistical analyses, confirm that the following items are present in the figure legend, table legend, main text, or Methods section.

n/a	Confirmed	
☐	☒	The exact sample size (*n*) for each experimental group/condition, given as a discrete number and unit of measurement
☐	☒	A statement on whether measurements were taken from distinct samples or whether the same sample was measured repeatedly
☐	☒	The statistical test(s) used AND whether they are one- or two-sided<br>*Only common tests should be described solely by name; describe more complex techniques in the Methods section.*
☒	☐	A description of all covariates tested
☒	☐	A description of any assumptions or corrections, such as tests of normality and adjustment for multiple comparisons
☐	☒	A full description of the statistical parameters including central tendency (e.g. means) or other basic estimates (e.g. regression coefficient) AND variation (e.g. standard deviation) or associated estimates of uncertainty (e.g. confidence intervals)
☐	☒	For null hypothesis testing, the test statistic (e.g. *F*, *t*, *r*) with confidence intervals, effect sizes, degrees of freedom and *P* value noted<br>*Give P values as exact values whenever suitable.*
☒	☐	For Bayesian analysis, information on the choice of priors and Markov chain Monte Carlo settings
☒	☐	For hierarchical and complex designs, identification of the appropriate level for tests and full reporting of outcomes
☒	☐	Estimates of effect sizes (e.g. Cohen's *d*, Pearson's *r*), indicating how they were calculated

Our web collection on statistics for biologists contains articles on many of the points above.

Software and code

Policy information about availability of computer code

Data collection	SBF-SEM data was collected using DigitalMicrograph Version 3.31 and Light microscopy data was collected using Nikon NIS Elements AR
Data analysis	All graphs and statistical analyses were performed using GraphPadPrism Version 9.4.1. or later. Image analysis was conducted with Fiji Version 2.9.0 and in Dragonfly software Version 2022.1.0.1259 for [Windows]

For manuscripts utilizing custom algorithms or software that are central to the research but not yet described in published literature, software must be made available to editors and reviewers. We strongly encourage code deposition in a community repository (e.g. GitHub). See the Nature Portfolio guidelines for submitting code & software for further information.

Data

Policy information about availability of data

All manuscripts must include a data availability statement. This statement should provide the following information, where applicable:

- Accession codes, unique identifiers, or web links for publicly available datasets
- A description of any restrictions on data availability
- For clinical datasets or third party data, please ensure that the statement adheres to our policy

Our SBF-SEM datasets are publicly available on EMPIAR-11683. Live-cell imaging and light microscopy data are available in Zenodo: 10.5281/zenodo.10009391.

Research involving human participants, their data, or biological material

Policy information about studies with human participants or human data. See also policy information about sex, gender (identity/presentation), and sexual orientation and race, ethnicity and racism.

Reporting on sex and gender	N/A
Reporting on race, ethnicity, or other socially relevant groupings	N/A
Population characteristics	N/A
Recruitment	N/A
Ethics oversight	N/A

Note that full information on the approval of the study protocol must also be provided in the manuscript.

Field-specific reporting

Please select the one below that is the best fit for your research. If you are not sure, read the appropriate sections before making your selection.

☒ Life sciences ☐ Behavioural & social sciences ☐ Ecological, evolutionary & environmental sciences

For a reference copy of the document with all sections, see nature.com/documents/nr-reporting-summary-flat.pdf

Life sciences study design

All studies must disclose on these points even when the disclosure is negative.

Sample size	For SBF-SEM datasets: For each infection time point (24 hpi and 48 hpi), we acquired two datasets, with each dataset containing a single infected cell. In addition, we acquired three datasets for subcellular regions of infected cells at 48 hpi, with each region containing at least one parasitophorous vacuole (PV). From all parasitophorous vacuoles across datasets, we were able to observe 64 sporonts, 61 sporoblasts and 140 spores. A minimum of 7 parasites were segmented completely per condition.<br>For light microscopy data: All quantified data statistics was performed on n>=3 biological replicates. For each biological replicate analysis was performed on n>= 50 cells per condition, per time point. The exception is live cell imaging data (and is noted in the figure legend) in which a total of 41 cells were analyzed from n=4 biological replicates.
Data exclusions	No data was excluded but for what we have noted in the methods section of segmentation analysis: We scored an additional 19 sporoblasts that are not included because they were hard to categorize clearly owing to imaging artifacts that preclude us from clearly identifying the PT across all imaged slices in these sporoblasts.
Replication	For all cell culture experiments replication was successful with at least three biological replicates per experiment
Randomization	Not applicable to cell culture experiments
Blinding	Blinding was not performed. Analyses of phenotypes in microscopy data were performed with clearly defined scoring criteria.

Reporting for specific materials, systems and methods

We require information from authors about some types of materials, experimental systems and methods used in many studies. Here, indicate whether each material, system or method listed is relevant to your study. If you are not sure if a list item applies to your research, read the appropriate section before selecting a response.

Materials & experimental systems

n/a	Involved in the study	
☐	☒	Antibodies
☐	☒	Eukaryotic cell lines
☒	☐	Palaeontology and archaeology
☒	☐	Animals and other organisms
☒	☐	Clinical data
☒	☐	Dual use research of concern
☒	☐	Plants

Methods

n/a	Involved in the study	
☒	☐	ChIP-seq
☒	☐	Flow cytometry
☒	☐	MRI-based neuroimaging

Antibodies

Antibodies used: Tom70 from proteintech (14528-1-AP); Goat anti-Rabbit IgG (H+L) Cross-Adsorbed Secondary Antibody, Alexa Fluor 488 (A11008)

Validation: Validated for immunofluorescence, western blot and immunoprecipitation by proteintech (14528-1-AP)

Eukaryotic cell lines

Policy information about cell lines and Sex and Gender in Research

Cell line source(s): Vero cells purchased from ATCC (ATCC® CCL-81™)

Authentication: Cell line has not been authenticated

Mycoplasma contamination: Cell lines tested negative for mycoplasma contamination

Commonly misidentified lines (See ICLAC register): No commonly misidentified cell lines have been used in this study
